# (*R*)-4-Phenyl-2-[(*S*)-1,2,3,4-tetra­hydro­isoquinolin-3-yl]-4,5-dihydro-1,3-oxazole

**DOI:** 10.1107/S1600536810022130

**Published:** 2010-06-26

**Authors:** Sai K. Chakka, Thavendran Govender, Hendrik G. Kruger, Glenn E. M. Maguire

**Affiliations:** a School of Chemistry, University of KwaZulu-Natal, Durban, South Africa; b School of Pharmacy and Pharmacology, University of KwaZulu-Natal, Durban, South Africa

## Abstract

The asymmetric unit cell of the title compound, C_18_H_18_N_2_O,  contains four molecules. In the crystal structure, an inter­molecular N—H⋯N hydrogen bond helps to establish the packing.

## Related literature

For the assymetric synthetic applications of oxazoline, see: Hargaden *et al.* (2009 ▶). For tetra­isoquinolines and their biological significance, see: Scott *et al.* (2002 ▶). For ligand catalysis activity, see: Chakka *et al.* (2010 ▶).
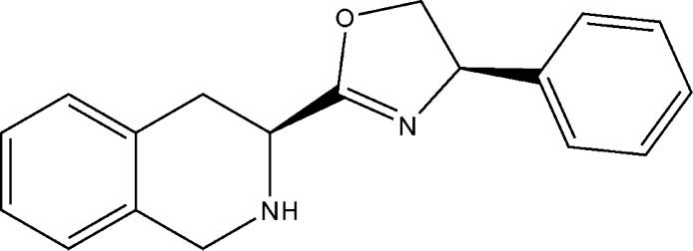


## Experimental

### 

#### Crystal data

C_18_H_18_N_2_O
*M* *_r_* = 278.34
Orthorhombic, 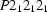

*a* = 5.4023 (3) Å
*b* = 10.0999 (6) Å
*c* = 26.2205 (17) Å
*V* = 1430.66 (15) Å^3^
*Z* = 4
Cu *K*α radiation
μ = 0.64 mm^−1^
*T* = 173 K
0.22 × 0.21 × 0.10 mm

#### Data collection

Bruker Kappa DUO APEXII diffractometer
Absorption correction: multi-scan (*SADABS*; Sheldrick, 1997 ▶) *T*_min_ = 0.682, *T*_max_ = 0.753
6634 measured reflections
1552 independent reflections
1479 reflections with *I* > 2σ(*I*)
*R*_int_ = 0.036

#### Refinement

*R*[*F* ^2^ > 2σ(*F* ^2^)] = 0.030
*wR*(*F* ^2^) = 0.076
*S* = 1.05
1552 reflections
195 parameters
1 restraint
H atoms treated by a mixture of independent and constrained refinement
Δρ_max_ = 0.14 e Å^−3^
Δρ_min_ = −0.12 e Å^−3^

### 

Data collection: *SAINT* (Bruker, 2006 ▶); cell refinement: *DENZO-SMN* (Otwinowski & Minor, 1997 ▶); data reduction: *DENZO-SMN*; program(s) used to solve structure: *SHELXS97* (Sheldrick, 2008 ▶); program(s) used to refine structure: *SHELXL97* (Sheldrick, 2008 ▶); molecular graphics: *X-SEED* (Barbour, 2001 ▶); software used to prepare material for publication: *SHELXL97*.

## Supplementary Material

Crystal structure: contains datablocks I, global. DOI: 10.1107/S1600536810022130/hg2694sup1.cif

Structure factors: contains datablocks I. DOI: 10.1107/S1600536810022130/hg2694Isup2.hkl

Additional supplementary materials:  crystallographic information; 3D view; checkCIF report

## Figures and Tables

**Table 1:** Hydrogen-bond geometry (Å, °)

*D*—H⋯*A*	*D*—H	H⋯*A*	*D*⋯*A*	*D*—H⋯*A*
N1—H1*N*⋯N2^i^	0.96 (1)	2.20 (1)	3.139 (2)	165 (2)

Symmetry code: (i) 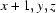.

**Fig. 1.**
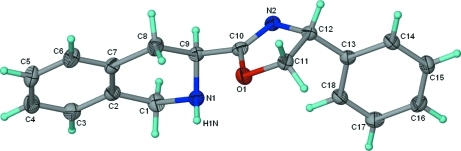
Molecular structure of (I) showing numbering scheme. All non-hydrogen atoms are shown as ellipsoids with probability level of 50%.

**Fig. 2.**
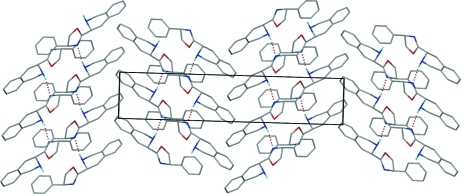
Projection viewed along [010]. All hydrogen atoms except the hydrogen H1N on N1 are omitted. The hydrogen bonds are shown as dotted lines.

## supplementary crystallographic information

### Comment

Heterocyclic rings play roles in a number of key areas of organic and inorganic chemistry. As part of an ongoing study into the asymmetric hydrogen transfer reactions we made a series of ligands (Chakka *et al.*, 2010). The title compound (I) is one such molecule (Fig 1). It combines for the first time two important heterocyclic rings into a single structure, namely oxazoline (Hargaden *et al.*, 2009) and tetraisoquinoline (TIQ), (Scott *et al.*, 2002). The asymmetric unit cell contains four molecules. There are inter-molecular N(1)—H···N(1) bond interactions (2.212 Å) that hold the structure in two dimensional planes. The intermolecular distance value between ring centroids in the *b *axis direction (5.402 Å), suggests that there is no π-stacking interaction between parallel molecules (Fig 2).

### Experimental

A solution of Cbz-protected TIQ*–*oxazoline (1.0 g, 2.89 mmol) in methanol (30 ml) was added to a suspension of 10 wt.% Pd/C (0.5 g) in methanol (10 ml). The reaction mixture was connected to an H_2_ source at atmospheric pressure and stirred at room temperature for 3 h. Completion of the reaction was monitored by TLC using hexane/ethyl acetate (7:3) with the *R*_f _= 0.6. The Pd/C was filtered off over a celite pad and the filtrate was concentrated under reduced pressure to afford crude (I). The title compound was purified on a deactivated silica gel column packed with a suspension of silica gel in 20% Et_3_N/CH_2_Cl_2_. The silica was washed with 1% Et_3_N/CH_2_Cl_2_. The chromatography was then performed using 0–2% MeOH/1%Et_3_N/CH_2_Cl_2_ as the eluent to afford TIQ-oxazoline product. *M*.p: 304 – 306 K.

^1^H NMR (400 MHz, CDCl_3_): *δ* 7.37–7.23 (m, 3H), 7.23–7.10 (m, 5H), 7.05 (m, 1H), 5.23 (t, *J* = 18.21 Hz, 1H), 4.68 (q, *J* = 10.10, 8.26 Hz, 1H), 4.16 (m, 3H), 3.92 (m, 1H), 3.10–3.05 (m, 2H); ^13^C NMR (100 MHz, CDCl_3_): *δ*168.9, 142.0, 135.0, 133.3, 129.2, 128.7, 127.6, 126.5, 126.2, 126.1, 126.0, 69.4, 51.5, 47.6, 32.4.

IR (neat): 3225, 1663, 1493, 1108, 957, 907, 916, 740, 749, 697 cm^-1^.

HR ESI MS: 279.1492 [*M* + H]^+^ (calcd. for C_18_H_19_N_2_O 279.1512).

### Refinement

All hydrogen atoms, except H1N on N1, were positioned geometrically with C—H = 0.95 - 1.00 Å and refined as riding on their parent atoms with *U*_iso_ (H) = 1.2 - 1.5 *U*_eq_ (C). The hydrogen atoms H1N were located in the difference electron density maps and refined with simple bond length constraint.

### Crystal data

**Table:**

C_18_H_18_N_2_O	*F*(000) = 592
*M**_r_* = 278.34	*D*_x_ = 1.292 Mg m^−^^3^
Orthorhombic, *P*2_1_2_1_2_1_	Cu *K*α radiation, λ = 1.54178 Å
Hall symbol: P 2ac 2ab	Cell parameters from 6634 reflections
*a* = 5.4023 (3) Å	θ = 4.7–69.2°
*b* = 10.0999 (6) Å	µ = 0.64 mm^−^^1^
*c* = 26.2205 (17) Å	*T* = 173 K
*V* = 1430.66 (15) Å^3^	Needle, colourless
*Z* = 4	0.22 × 0.21 × 0.10 mm

### Data collection

**Table:**

Bruker Kappa DUO APEXII diffractometer	1552 independent reflections
Radiation source: fine-focus sealed tube	1479 reflections with *I* > 2σ(*I*)
graphite	*R*_int_ = 0.036
Detector resolution: n/a pixels mm^-1^	θ_max_ = 69.2°, θ_min_ = 4.7°
0.5° φ scans and ω scans	*h* = −6→6
Absorption correction: multi-scan (*SADABS*; Sheldrick, 1997)	*k* = −11→12
*T*_min_ = 0.682, *T*_max_ = 0.753	*l* = −15→30
6634 measured reflections	

### Refinement

**Table:**

Refinement on *F*^2^	Secondary atom site location: difference Fourier map
Least-squares matrix: full	Hydrogen site location: inferred from neighbouring sites
*R*[*F*^2^ > 2σ(*F*^2^)] = 0.030	H atoms treated by a mixture of independent and constrained refinement
*wR*(*F*^2^) = 0.076	*w* = 1/[σ^2^(*F*_o_^2^) + (0.0436*P*)^2^ + 0.1838*P*] where *P* = (*F*_o_^2^ + 2*F*_c_^2^)/3
*S* = 1.05	(Δ/σ)_max_ < 0.001
1552 reflections	Δρ_max_ = 0.14 e Å^−^^3^
195 parameters	Δρ_min_ = −0.12 e Å^−^^3^
1 restraint	Extinction correction: *SHELXL97* (Sheldrick, 2008), Fc^*^=kFc[1+0.001xFc^2^λ^3^/sin(2θ)]^-1/4^
Primary atom site location: structure-invariant direct methods	Extinction coefficient: 0.0039 (5)

### Special details

**Table:**

Experimental. Half sphere of data collected using *SAINT* strategy (Bruker, 2006). Crystal to detector distance = 50 mm; combination of φ and ω scans of 0.5°, 30 s per °, 2 iterations.
Geometry. All e.s.d.'s (except the e.s.d. in the dihedral angle between two l.s. planes) are estimated using the full covariance matrix. The cell e.s.d.'s are taken into account individually in the estimation of e.s.d.'s in distances, angles and torsion angles; correlations between e.s.d.'s in cell parameters are only used when they are defined by crystal symmetry. An approximate (isotropic) treatment of cell e.s.d.'s is used for estimating e.s.d.'s involving l.s. planes.
Refinement. Refinement of *F*^2^ against ALL reflections. The weighted *R*-factor *wR* and goodness of fit *S* are based on *F*^2^, conventional *R*-factors *R* are based on *F*, with *F* set to zero for negative *F*^2^. The threshold expression of *F*^2^ > σ(*F*^2^) is used only for calculating *R*-factors(gt) *etc*. and is not relevant to the choice of reflections for refinement. *R*-factors based on *F*^2^ are statistically about twice as large as those based on *F*, and *R*- factors based on ALL data will be even larger.

### Fractional atomic coordinates and isotropic or equivalent isotropic displacement parameters (Å^2^)

**Table:**

	*x*	*y*	*z*	*U*_iso_*/*U*_eq_	
O1	0.4184 (2)	0.79986 (13)	0.19408 (5)	0.0306 (3)	
N1	0.5780 (3)	1.06125 (15)	0.15598 (6)	0.0272 (3)	
H1N	0.702 (3)	1.0028 (18)	0.1694 (7)	0.033 (6)*	
N2	0.0533 (3)	0.90911 (15)	0.19216 (6)	0.0285 (4)	
C1	0.6813 (4)	1.15005 (18)	0.11756 (7)	0.0324 (4)	
H1A	0.8214	1.1991	0.1329	0.039*	
H1B	0.5533	1.2157	0.1080	0.039*	
C2	0.7714 (3)	1.08180 (18)	0.06969 (7)	0.0269 (4)	
C3	0.9535 (4)	1.13952 (18)	0.03901 (7)	0.0322 (4)	
H3	1.0184	1.2238	0.0480	0.039*	
C4	1.0408 (4)	1.0762 (2)	−0.00419 (7)	0.0363 (5)	
H4	1.1656	1.1162	−0.0245	0.044*	
C5	0.9444 (4)	0.9538 (2)	−0.01748 (7)	0.0382 (5)	
H5	1.0025	0.9096	−0.0471	0.046*	
C6	0.7634 (4)	0.89605 (19)	0.01249 (7)	0.0356 (5)	
H6	0.6978	0.8123	0.0031	0.043*	
C7	0.6755 (4)	0.95876 (18)	0.05633 (7)	0.0285 (4)	
C8	0.4854 (4)	0.89025 (19)	0.08944 (7)	0.0350 (5)	
H8A	0.5606	0.8097	0.1043	0.042*	
H8B	0.3448	0.8621	0.0678	0.042*	
C9	0.3869 (3)	0.97793 (18)	0.13282 (7)	0.0272 (4)	
H9	0.2567	1.0374	0.1183	0.033*	
C10	0.2691 (3)	0.89535 (18)	0.17416 (7)	0.0260 (4)	
C11	0.2612 (3)	0.72424 (17)	0.22836 (7)	0.0279 (4)	
H11A	0.3444	0.7089	0.2615	0.033*	
H11B	0.2159	0.6378	0.2132	0.033*	
C12	0.0312 (3)	0.81315 (17)	0.23493 (7)	0.0258 (4)	
H12	−0.1226	0.7592	0.2308	0.031*	
C13	0.0251 (3)	0.88531 (17)	0.28547 (6)	0.0247 (4)	
C14	−0.1619 (3)	0.86228 (18)	0.32043 (7)	0.0288 (4)	
H14	−0.2902	0.8014	0.3124	0.035*	
C15	−0.1639 (4)	0.92738 (19)	0.36719 (7)	0.0327 (4)	
H15	−0.2932	0.9106	0.3909	0.039*	
C16	0.0209 (4)	1.01606 (19)	0.37932 (7)	0.0329 (4)	
H16	0.0188	1.0608	0.4112	0.040*	
C17	0.2105 (4)	1.03964 (19)	0.34458 (7)	0.0330 (4)	
H17	0.3394	1.0999	0.3528	0.040*	
C18	0.2114 (3)	0.97527 (18)	0.29802 (7)	0.0290 (4)	
H18	0.3403	0.9925	0.2743	0.035*	

### Atomic displacement parameters (Å^2^)

**Table:**

	*U*^11^	*U*^22^	*U*^33^	*U*^12^	*U*^13^	*U*^23^
O1	0.0268 (6)	0.0313 (6)	0.0336 (7)	0.0056 (6)	0.0025 (5)	0.0064 (5)
N1	0.0279 (7)	0.0272 (7)	0.0267 (7)	0.0012 (7)	0.0002 (6)	−0.0021 (6)
N2	0.0249 (7)	0.0349 (8)	0.0257 (7)	0.0035 (7)	−0.0025 (6)	0.0027 (7)
C1	0.0363 (11)	0.0260 (8)	0.0349 (10)	−0.0015 (8)	0.0026 (8)	−0.0034 (8)
C2	0.0281 (9)	0.0266 (8)	0.0260 (8)	0.0031 (8)	−0.0035 (7)	0.0029 (7)
C3	0.0335 (10)	0.0292 (9)	0.0338 (10)	−0.0020 (8)	−0.0014 (8)	0.0045 (8)
C4	0.0385 (10)	0.0396 (10)	0.0309 (9)	0.0013 (10)	0.0044 (8)	0.0087 (8)
C5	0.0493 (12)	0.0387 (10)	0.0267 (9)	0.0046 (10)	0.0070 (9)	0.0009 (8)
C6	0.0488 (12)	0.0302 (9)	0.0279 (9)	−0.0025 (9)	0.0006 (8)	−0.0024 (7)
C7	0.0326 (9)	0.0291 (9)	0.0237 (8)	0.0016 (8)	−0.0018 (7)	0.0029 (7)
C8	0.0437 (12)	0.0335 (9)	0.0277 (9)	−0.0099 (9)	0.0030 (8)	−0.0035 (8)
C9	0.0253 (9)	0.0310 (9)	0.0252 (9)	0.0017 (8)	−0.0027 (7)	0.0016 (7)
C10	0.0260 (9)	0.0272 (8)	0.0247 (8)	0.0024 (8)	−0.0050 (7)	−0.0001 (7)
C11	0.0297 (9)	0.0262 (8)	0.0278 (9)	0.0022 (8)	0.0005 (7)	0.0006 (7)
C12	0.0227 (8)	0.0284 (8)	0.0262 (8)	0.0007 (7)	−0.0006 (7)	−0.0002 (8)
C13	0.0232 (8)	0.0245 (8)	0.0264 (8)	0.0045 (7)	−0.0024 (7)	0.0029 (7)
C14	0.0254 (9)	0.0281 (9)	0.0329 (10)	−0.0005 (8)	0.0017 (8)	0.0027 (7)
C15	0.0311 (10)	0.0358 (10)	0.0313 (9)	0.0013 (9)	0.0059 (8)	0.0021 (8)
C16	0.0360 (11)	0.0361 (9)	0.0267 (8)	0.0044 (9)	−0.0025 (8)	−0.0049 (8)
C17	0.0320 (9)	0.0301 (9)	0.0368 (10)	−0.0044 (8)	−0.0050 (8)	−0.0035 (8)
C18	0.0244 (9)	0.0310 (9)	0.0316 (9)	−0.0010 (8)	0.0011 (7)	0.0030 (8)

### Geometric parameters (Å, °)

**Table:**

O1—C10	1.362 (2)	C8—C9	1.537 (2)
O1—C11	1.454 (2)	C8—H8A	0.9900
N1—C1	1.460 (2)	C8—H8B	0.9900
N1—C9	1.464 (2)	C9—C10	1.508 (2)
N1—H1N	0.960 (10)	C9—H9	1.0000
N2—C10	1.265 (2)	C11—C12	1.542 (3)
N2—C12	1.487 (2)	C11—H11A	0.9900
C1—C2	1.513 (2)	C11—H11B	0.9900
C1—H1A	0.9900	C12—C13	1.513 (2)
C1—H1B	0.9900	C12—H12	1.0000
C2—C7	1.391 (3)	C13—C14	1.384 (2)
C2—C3	1.398 (3)	C13—C18	1.395 (3)
C3—C4	1.384 (3)	C14—C15	1.391 (3)
C3—H3	0.9500	C14—H14	0.9500
C4—C5	1.386 (3)	C15—C16	1.378 (3)
C4—H4	0.9500	C15—H15	0.9500
C5—C6	1.383 (3)	C16—C17	1.391 (3)
C5—H5	0.9500	C16—H16	0.9500
C6—C7	1.396 (3)	C17—C18	1.383 (3)
C6—H6	0.9500	C17—H17	0.9500
C7—C8	1.512 (3)	C18—H18	0.9500
			
C10—O1—C11	105.26 (13)	C10—C9—C8	111.06 (15)
C1—N1—C9	109.66 (14)	N1—C9—H9	108.0
C1—N1—H1N	111.3 (13)	C10—C9—H9	108.0
C9—N1—H1N	106.9 (13)	C8—C9—H9	108.0
C10—N2—C12	106.47 (15)	N2—C10—O1	118.72 (17)
N1—C1—C2	114.56 (14)	N2—C10—C9	126.58 (17)
N1—C1—H1A	108.6	O1—C10—C9	114.67 (15)
C2—C1—H1A	108.6	O1—C11—C12	103.52 (14)
N1—C1—H1B	108.6	O1—C11—H11A	111.1
C2—C1—H1B	108.6	C12—C11—H11A	111.1
H1A—C1—H1B	107.6	O1—C11—H11B	111.1
C7—C2—C3	119.32 (17)	C12—C11—H11B	111.1
C7—C2—C1	119.75 (16)	H11A—C11—H11B	109.0
C3—C2—C1	120.92 (17)	N2—C12—C13	110.39 (13)
C4—C3—C2	121.21 (18)	N2—C12—C11	103.33 (14)
C4—C3—H3	119.4	C13—C12—C11	113.32 (14)
C2—C3—H3	119.4	N2—C12—H12	109.9
C3—C4—C5	119.33 (19)	C13—C12—H12	109.9
C3—C4—H4	120.3	C11—C12—H12	109.9
C5—C4—H4	120.3	C14—C13—C18	118.68 (17)
C6—C5—C4	119.93 (19)	C14—C13—C12	121.02 (16)
C6—C5—H5	120.0	C18—C13—C12	120.29 (15)
C4—C5—H5	120.0	C13—C14—C15	120.66 (17)
C5—C6—C7	121.13 (19)	C13—C14—H14	119.7
C5—C6—H6	119.4	C15—C14—H14	119.7
C7—C6—H6	119.4	C16—C15—C14	120.32 (18)
C2—C7—C6	119.08 (18)	C16—C15—H15	119.8
C2—C7—C8	121.13 (16)	C14—C15—H15	119.8
C6—C7—C8	119.76 (17)	C15—C16—C17	119.57 (17)
C7—C8—C9	113.36 (15)	C15—C16—H16	120.2
C7—C8—H8A	108.9	C17—C16—H16	120.2
C9—C8—H8A	108.9	C18—C17—C16	119.98 (18)
C7—C8—H8B	108.9	C18—C17—H17	120.0
C9—C8—H8B	108.9	C16—C17—H17	120.0
H8A—C8—H8B	107.7	C17—C18—C13	120.78 (17)
N1—C9—C10	108.50 (14)	C17—C18—H18	119.6
N1—C9—C8	113.21 (15)	C13—C18—H18	119.6
			
C9—N1—C1—C2	−52.7 (2)	C11—O1—C10—C9	−173.78 (14)
N1—C1—C2—C7	23.6 (3)	N1—C9—C10—N2	108.6 (2)
N1—C1—C2—C3	−155.27 (17)	C8—C9—C10—N2	−126.4 (2)
C7—C2—C3—C4	−0.4 (3)	N1—C9—C10—O1	−69.47 (19)
C1—C2—C3—C4	178.49 (18)	C8—C9—C10—O1	55.6 (2)
C2—C3—C4—C5	0.5 (3)	C10—O1—C11—C12	−14.48 (17)
C3—C4—C5—C6	−0.3 (3)	C10—N2—C12—C13	109.57 (16)
C4—C5—C6—C7	−0.2 (3)	C10—N2—C12—C11	−11.91 (18)
C3—C2—C7—C6	−0.1 (3)	O1—C11—C12—N2	15.96 (17)
C1—C2—C7—C6	−178.97 (17)	O1—C11—C12—C13	−103.52 (16)
C3—C2—C7—C8	177.66 (17)	N2—C12—C13—C14	127.42 (17)
C1—C2—C7—C8	−1.2 (3)	C11—C12—C13—C14	−117.22 (18)
C5—C6—C7—C2	0.4 (3)	N2—C12—C13—C18	−53.6 (2)
C5—C6—C7—C8	−177.41 (18)	C11—C12—C13—C18	61.8 (2)
C2—C7—C8—C9	8.5 (3)	C18—C13—C14—C15	−0.1 (3)
C6—C7—C8—C9	−173.72 (17)	C12—C13—C14—C15	178.90 (16)
C1—N1—C9—C10	−175.40 (15)	C13—C14—C15—C16	0.1 (3)
C1—N1—C9—C8	60.83 (19)	C14—C15—C16—C17	−0.3 (3)
C7—C8—C9—N1	−38.5 (2)	C15—C16—C17—C18	0.7 (3)
C7—C8—C9—C10	−160.89 (15)	C16—C17—C18—C13	−0.7 (3)
C12—N2—C10—O1	3.0 (2)	C14—C13—C18—C17	0.4 (3)
C12—N2—C10—C9	−174.98 (16)	C12—C13—C18—C17	−178.58 (17)
C11—O1—C10—N2	8.0 (2)		

### Hydrogen-bond geometry (Å, °)

**Table:**

*D*—H···*A*	*D*—H	H···*A*	*D*···*A*	*D*—H···*A*
N1—H1N···N2^i^	0.96 (1)	2.20 (1)	3.139 (2)	165 (2)

Symmetry codes: (i) *x*+1, *y*, *z*.

## References

[bb1] Barbour, L. J. (2001). *J. Supramol. Chem.* **1**, 189–191.

[bb2] Bruker (2006). *SAINT* Bruker AXS Inc., Madison, Wisconsin, USA.

[bb3] Chakka, S. K., Andersson, P. G., Maguire, G. E. M., Kruger, H. G. & Govender, T. (2010). *Eur. J. Org. Chem.* pp. 972–980.

[bb4] Hargaden, G. C. & Guiry, P. J. (2009). *Chem. Rev.* **109**, 2505–2550. 10.1021/cr800400z 19378971

[bb5] Otwinowski, Z. & Minor, W. (1997). *Methods in Enzymology*, Vol. 276, *Macromolecular Crystallography*, Part A, edited by C. W. Carter Jr & R. M. Sweet, pp. 307–326. New York: Academic Press.

[bb6] Scott, J. D. & Williams, R. M. (2002). *Chem. Rev.* **102**, 1669–1730. 10.1021/cr010212u 11996547

[bb7] Sheldrick, G. M. (1997). *SADABS* University of Göttingen, Germany.

[bb8] Sheldrick, G. M. (2008). *Acta Cryst.* A**64**, 112–122. 10.1107/S0108767307043930 18156677

